# Ginseng for managing menopausal woman's health: A systematic review of double-blind, randomized, placebo-controlled trials

**DOI:** 10.1097/MD.0000000000004914

**Published:** 2016-09-23

**Authors:** Hye Won Lee, Jiae Choi, YoungJoo Lee, Ki-Jung Kil, Myeong Soo Lee

**Affiliations:** a KM Convergence Research Division; b Clinical Research Division, Korea Institute of Oriental Medicine, Daejeon; c Department of Bioscience and Biotechnology, College of Life Science, Sejong University, Seoul; d College of Oriental Medicine, Joongbu University, Chungnam, Republic of Korea; e Allied Health Sciences, London South Bank University, London, UK.

**Keywords:** complementary medicine, ginseng, menopause symptoms, women's health

## Abstract

**Background:**

The aim of this systematic review was to update, complete, and critically evaluate the evidence from placebo-controlled randomized clinical trials (RCTs) of ginseng for managing menopausal women's health.

**Methods:**

We searched the literature using 13 databases (MEDLINE, AMED, EMBASE, the Cochrane Library, 6 Korean Medical, and 3 Chinese Databases) from their inception to July 2016 and included all double-blind RCTs that compared any type of ginseng with a placebo control in postmenopausal women. The methodological quality of all studies was assessed using a Cochrane risk of bias tool.

**Results:**

Ten RCTs met our inclusion criteria. Most RCTs had unclear risk of bias. One RCT did not show a significant difference in hot flash frequency between Korean red ginseng (KRG) and placebo. The second RCT reported positive effects of KRG on menopausal symptoms. The third RCT found beneficial effects of ginseng (Ginsena) on depression, well-being, and general health. Four RCTs failed to show significant differences in various hormones between KRG and placebo controls except dehydroepiandrosterone. Two other RCTs failed to show effects of KRG on endometrial thickness in menopausal women. The other RCT also failed to show the effects of American ginseng on oxidative stress markers and other antioxidant enzymes.

**Conclusion:**

Our systematic review provided positive evidence of ginseng for sexual function and KRG for sexual arousal and total hot flashes score in menopausal women. However, the results of KRG or ginseng failed to show specific effects on hot flash frequency, hormones, biomarkers, or endometrial thickness. The level of evidence for these findings was low because of unclear risk of bias.

## 1 Introduction

Menopausal women experience mood changes, hot flashes, sleeplessness, vaginal dryness, night sweats, decreased libido, and impairment of cognitive function.^[1]^ Some of these symptoms can be effectively treated with hormone replacement therapy (HRT). However, the risks associated with HRT lead many menopausal women to use complementary therapies. One systematic review showed that 32.9% of menopausal women used CAM, and 47.7% had used CAM in the last 12 months.^[2]^ Herbal medicines are the most popular form of CAM, reportedly used by 34.6% of women. This review finds that ginseng is one of the frequently used single herbs for menopausal symptoms.^[2]^

Ginseng has been used to improve overall health, reduce stress, boost energy, and enhance the immune system.^[3]^ Ginseng is said to function as an “adaptogen,”^[4]^ an agent that promotes resistance to external and internal stresses and improves both physical and mental faculties. Reports in the literature have suggested that ginseng may offer other benefits, including reduced risk of certain cancers, hypertension, and diabetes, improved sexual function, and reduced risks of the common cold. The evidence on ginseng for managing menopausal symptoms is limited.

There is 1 systematic review of ginseng for menopausal symptoms.^[5]^ It included 4 randomized clinical trials (RCTs) of ginseng compared with a placebo control. This review suggested that ginseng may be beneficial for menopausal symptom management, but it is needed to provide additional evidences from recently published trials.

Therefore, the aim of this systematic review was to update, complete, and critically evaluate the evidence from placebo-controlled RCTs of ginseng for managing menopausal women's health.

## 2 Methods

This study did not have the ethical approval because our data for this study were analyzed from the published primary studies.

### 2.1 Data sources

The following databases were searched from their inception to July 2016: MEDLINE, AMED, EMBASE, the Cochrane Library, 6 Korean Medical Databases (Korean Studies Information Service System, DBPIA, the Korean Institute of Science and Technology Information, the Research Information Service System, KoreaMed, and the Korean National Assembly Library), the China National Knowledge Infrastructure (CNKI), the Chongqing VIP Chinese Science and Technology Periodical (VIP), and Wanfang. Articles identified through reference lists of included studies and relevant systematic reviews were also considered for inclusion. The search terms used were “(ginseng OR ginseng$ OR panaxa) AND (menopause$ OR climact$ OR perimenopaus$ OR peri-menopaus$ OR post menopause$ OR post-menopaus$ OR hot flash$ OR hot-flash$ OR hot flush$ OR hot-flush$).” In addition, our own files and a relevant journal (FACT—*Focus on Alternative and Complementary Therapies*) were manually searched.

### 2.2 Types of studies

Randomized and quasi-randomized placebo controlled trials were included.

### 2.3 Types of participants

We included trials with the following types of participants.

Menopausal women were included. We excluded trials of patients with breast cancer, endometriosis, or those with patients who are immuno-compromised or taking multiple medications.

### 2.4 Types of interventions

All prospective randomized clinical studies that compared the effects of any type of ginseng on menopause symptoms with those of a placebo were included. The trials included extracts of Korean ginseng (*Panax ginseng*), Chinese ginseng (*Panax notoginseng*) or American ginseng (*Panax quinquefolius*), or commercial products made from Korean ginseng or American ginseng, regardless of age, processing status (e.g., fresh ginseng, white ginseng, or red ginseng), or dose. We compared placebo controls to ginseng therapies used alone or in combination with other conventional treatments. Trials in which ginseng formed part of a complex herbal medicine were excluded. Dissertations and abstracts were also included.

### 2.5 Types of outcome measures

#### 2.5.1 Primary outcomes

1. Total treatment efficacy: the number of patients whose menopausal symptoms were improved
2. Menopausal symptoms: measured by Kupperman index and other questionnaires for measuring menopausal symptoms

#### 2.5.2 Secondary outcomes

1. Quality of life
2. Hormones, biochemical parameters

### 2.6 Data extraction

All articles were read by 2 independent reviewers who extracted data from the articles according to predefined criteria. The extracted data included the authors, year of publication, country, sample size, age of the participants, types of ginseng, dose, treatment duration, main outcomes, and adverse effects. The extracted data were tabulated for analysis. No language restrictions were imposed.

### 2.7 Risk of bias assessment

Risk of bias (ROB) was assessed with the Cochrane ROB criteria: random sequence generation, allocation concealment, blinding of participants and personnel, blinding of outcome assessment, incomplete outcome data, selective outcome reporting, and other sources of bias (we will evaluate baseline imbalance).^[6]^ This review will use “L, U and H” as a key for these judgments, where “Low” (L) indicates a low ROB, “Unclear” U indicates that the ROB is uncertain, and “High” (H) indicates a high ROB. Disagreements were resolved by discussion among all authors. The ROB assessment for the included studies is summarized in a table, and the results and implications were critically discussed.

### 2.8 Data synthesis

All statistical analyses were conducted using the Cochrane Collaboration's software program Review Manager (RevMan), V.5.3 for Windows (Copenhagen, The Nordic Cochrane Centre). Differences between the intervention and placebo control groups were assessed. In the analysis of clinical efficacy, categorical data were assessed in terms of risk ratios, and continuous data were assessed in terms of mean difference (MD). Categorical and continuous variables were expressed as efficacy values with 95% confidence intervals (CIs). In cases of outcome variables with different scales, the standardized MD was used instead of the weighted MD. If the meta-analysis exhibited heterogeneity (defined as results of tests of heterogeneity that indicate a value of *P* < 0.1 by Chi-square test and Higgins I^2^ ≥ 50%), subgroup analyses were explored to determine the cause of the clinical heterogeneity. A random effects model was used to assess combined effect size from efficacy variables because clinical heterogeneity is highly expected across the included studies from the diversity of interventions, study design, and other conditions. Publication bias was assessed using funnel plots and Egger regression method.^[7]^ If missing data were detected, we requested any missing or incomplete information from the original study investigators. Subgroup analyses were conducted according to the type of ginseng, dose, and treatment duration. Where appropriate, sensitivity analysis was performed to evaluate the robustness of the meta-analysis results.

## 3 Results

**Figure 1:**
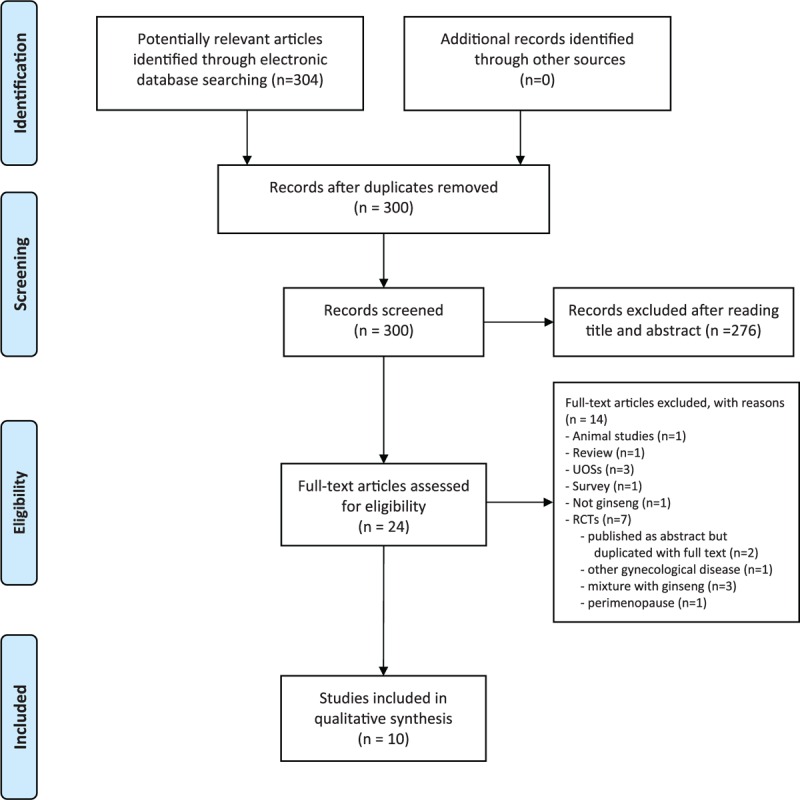
Flowchart of trial selection process. CCT = controlled clinical trial, RCT = randomized clinical trial, UOS = uncontrolled observational study.

The search revealed 304 possibly relevant studies. Seven RCTs were excluded for the reasons given in Fig. 1. Key data from the remaining 10 RCTs are summarized in Table 1.^[8–17]^ Eight of the included studies were performed in Korea,^[8,9,11–16]^ 1 trial was from Sweden,^[10]^ and 1 study was from USA.^[17]^ Six trials included the postmenopausal women who had not menstruated naturally from 6 to 12 months,^[8–11,14,16]^ while the other 4 did not report in details.^[12,13,15,17]^ Seven trials used Korean red ginseng (KRG),^[8,9,11,13–16]^ 1 study employed fermented KRG,^[12]^ and the other 2 used American ginseng (Ginsena and another commercial supplement). Eight RCTs adopted a 2-arm parallel group design,^[8–10,12,14–17]^ and 2 used a cross-over design.^[11,13]^ The doses of ginseng ranged from 200 to 3000 mg per day, and the treatment ranged from 2 to 16 weeks.

**Table 1:**
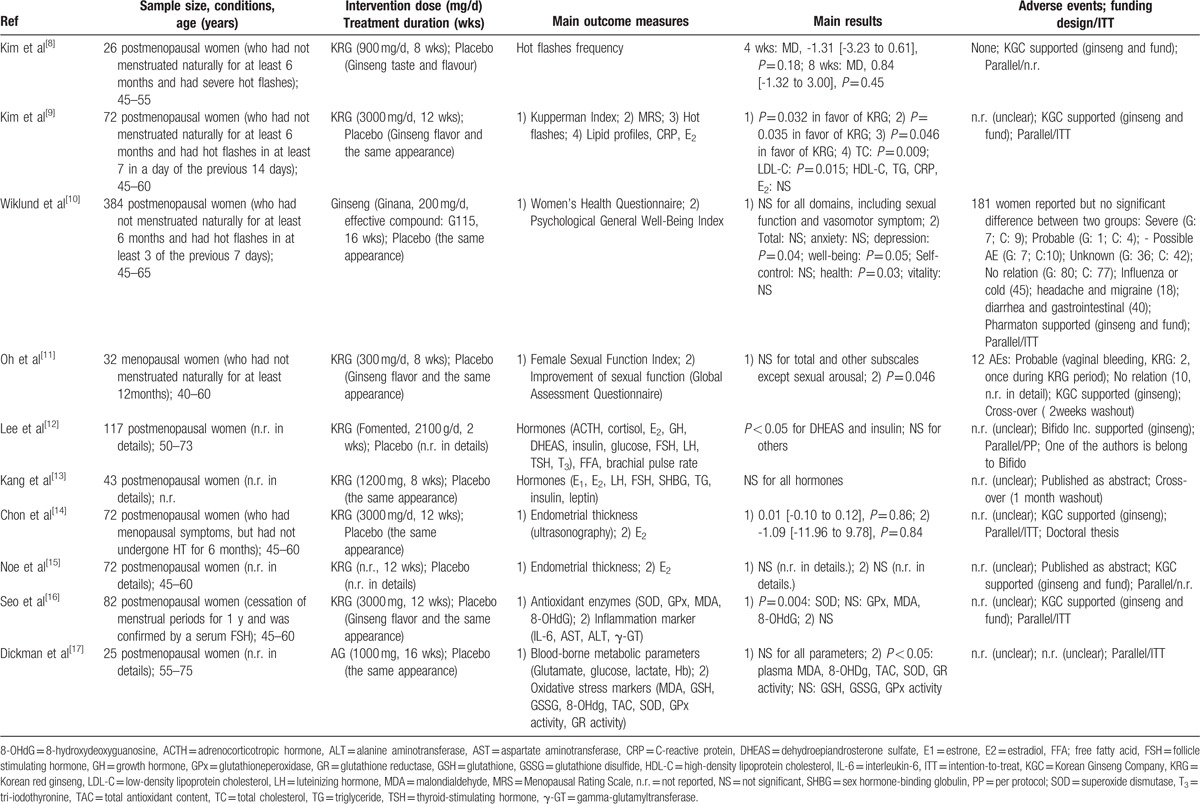
Summary of double-blind, randomized, placebo-controlled trials of ginseng for menopausal women's health.

### 3.1 Risk of bias

**Figure 2:**
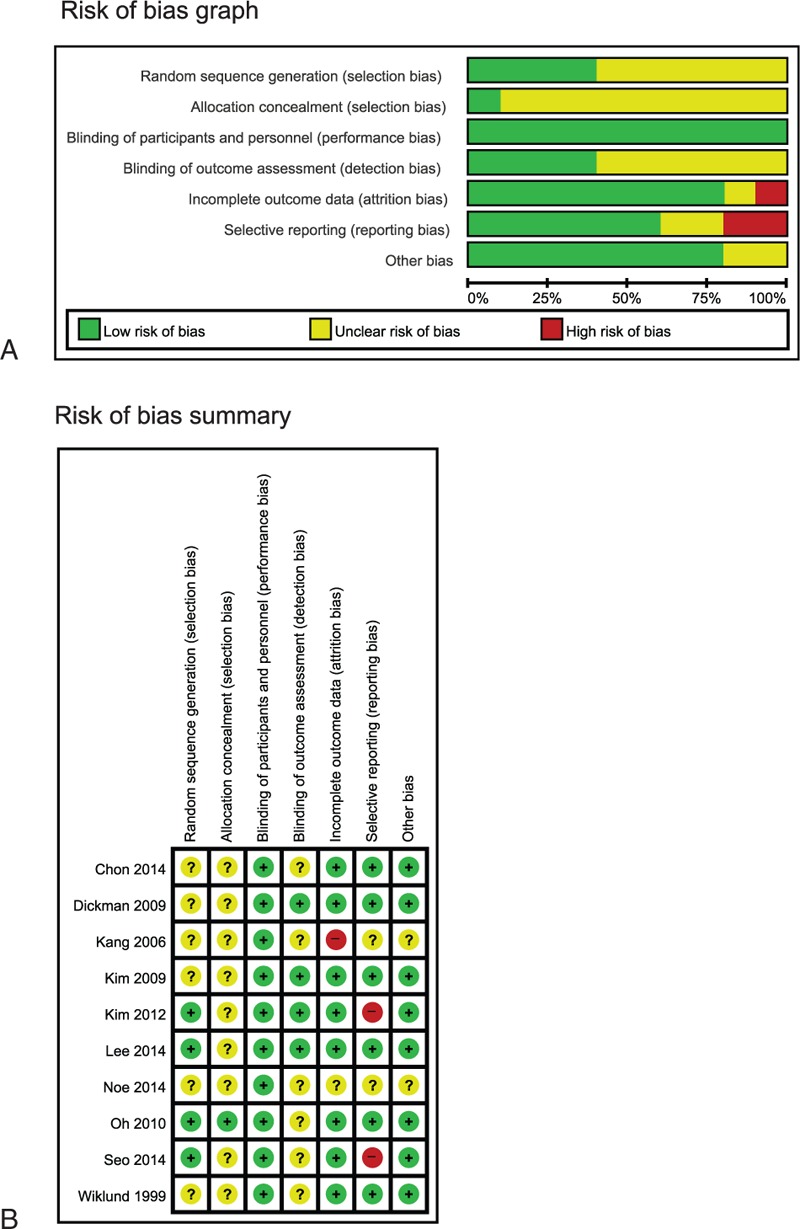
(A) Risk of bias graph: review authors’ judgments about each item's risk of bias item presented as percentage across all included studies; (B) Risk of bias summary: review authors’ judgments about each item's risk of bias for each included study. (+): low risk of bias; (−): high risk of bias; (?): unclear.

Most trials had a moderate ROB (Fig. 2). Only 4 employed an adequate sequence generation method for randomization,^[9,11,12,16]^ while the other 6 did not report it.^[8,10,13–15,17]^ All of the included trials used patient and therapist blinding, but only 3 RCTs adopted assessor blinding.^[8,9,12]^ None of the trials employed allocation concealment. Eight RCTs had a low ROB in incomplete outcomes,^[8–12,14,16,17]^ but the other 2 studies,^[13,15]^ which were published in abstract only, had an unclear^[15]^ or high ROB.^[13]^ Six RCTs had a low ROB in selective reporting^[8,10–12,14,17]^; 2 trials had an unclear ROB,^[13,15]^ and 2 had a high ROB.^[9,16]^ Only 5 RCTs noted using an intention-to-treat (ITT) analysis.^[9,10,14,16,17]^

### 3.2 Outcomes

#### 3.2.1 Menopausal symptoms

**Figure 3:**
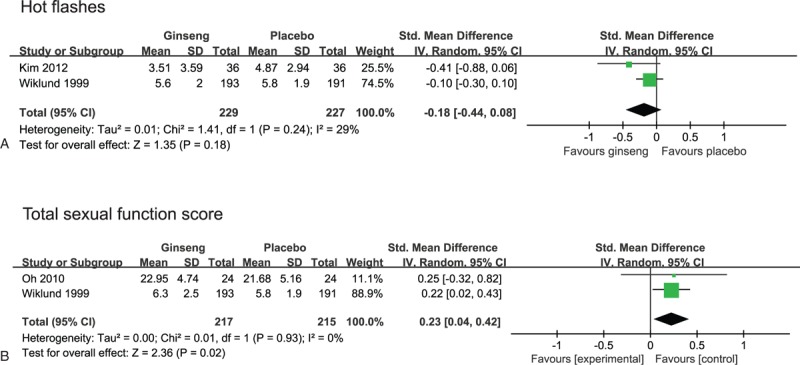
Forest plot of ginseng on (A) hot flashes; (B) total sexual function score.

Three RCTs tested the efficacy of KRG or ginseng for menopausal symptoms including hot flashes.^[8–10]^ One RCT did not show a significant difference in hot flash frequency between KRG and placebo.^[8]^ The second RCT reported positive effects of KRG on menopausal symptoms using Kupperman Index and the Menopause Rating Scale and improvement of hot flashes.^[9]^ The third RCT compared a commercial ginseng preparation (Ginsena) with a placebo for menopause symptoms and found beneficial effects of ginseng on depression, well-being, and general health, while it failed to do so for vasomotor symptom improvement.^[10]^ One RCT showed positive effects of KRG on hot flashes,^[9]^ while the other RCT failed to find an effect of ginseng on vasomotor symptoms.^[8,10]^ The meta-analysis failed to show favorable effects of ginseng on vasomotor symptoms (n = 459, SMD, -0.18, 95%CIs: -0.44 to 0.08, *P* = 0.18, I^2^ = 29%, Fig. 3A).

#### 3.2.2 Sexual function

Two RCTs investigated the efficacy of ginseng for sexual function in pre- and postmenopausal women.^[10,11]^ One RCT failed to show effects of ginseng on sexual function.^[10]^ The second RCT did not report favorable effects of KRG on sexual functions except for arousal subscale and improvement of sexual function in postmenopausal women.^[11]^ The meta-analysis of 2 studies showed favorable effects of ginseng for sexual function score (n = 432, SMD, 0.23, 95% CIs: 0.04–0.42, *P* = 0.02, I^2^ = 0%, Fig. 3B).

#### 3.2.3 Hormone level

Three trials tested the effects of KRG on hormone levels, including testosterone, E2, luteinizing hormone (LH), follicle-stimulating hormone (FSH), and others.^[9,12,13]^ All 4 of the included RCTs failed to show significant differences in various hormones between KRG and placebo controls except DHEA.^[12]^

#### 3.2.4 Other biochemical variables

One RCT assessed the antioxidative effects of KRG compared with a placebo. The results showed positive effects of KRG on superoxide dismutase (SOD), while they failed to do so for other antioxidant enzymes, including glutathione peroxidase, malondialdehyde, and 8-hydroxydeoxyguanosine.^[16]^ The other RCT failed to show the effects of American ginseng on oxidative stress markers and other antioxidant enzymes.^[17]^

#### 3.2.5 Endometrial thickness

Two RCTs assessed the effects of KRG on endometrial thickness and E2 level.^[14,15]^ Both RCTs failed to show effects of KRG on endometrial thickness in menopausal women.

#### 3.2.6 Adverse events

Three RCTs assessed adverse events (AEs),^[8,10,11]^ while the other 7 studies did not. One RCT noted no AEs.^[8]^ The second RCT reported the most frequent AEs, including influenza and common cold (45), headache and migraine (18), and diarrhea and other gastrointestinal symptoms (40) in 181 of 384 participants without a significant difference between the 2 groups.^[10]^ The third RCT noted 12 AEs including vaginal bleeding (2), which was most likely related to KRG use, but the other 10 AEs were unrelated to ginseng.^[11]^

## 4 Discussion

Few rigorous RCTs have assessed the efficacy of ginseng for menopausal women's health. Our systematic review provided suggestive evidence of ginseng for sexual function and sexual arousal in menopausal women. However, the results failed to show specific effects of ginseng on vasomotor symptoms, hormones, biomarkers, and endometrial thickness. The level of evidence for these findings was low because of unclear ROB. Furthermore, the number of trials and total sample size of the included trials were not sufficient to draw firm conclusions.

Although all of the 11 RCTs were double blinded, only 4 reported random sequence generation,^[9,11,12,16]^ while all of the included studies suffered from a lack of adequate allocation concealment. Trials with inadequate blinding and inadequate allocation concealment are likely to show exaggerated treatment effects.^[18]^ Only 3 trials used assessor blinding when appropriate,^[8,9,12]^ and 5 trials used an ITT analysis.^[9,10,14,16,17]^ Two were published as an abstract, 1 was a doctoral thesis^[14]^; thus, they did not undergo a peer review process. Two studies,^[14,15]^ which tested the effects of KRG on endometrial thickness, seem to be the same study. One study was as a published doctoral dissertation,^[14]^ and the other was in abstract only.^[15]^ They had the same characteristics of participants and outcomes except different authors. Even if this is true, it would not influence our conclusion.

Although all of the RCTs used placebo controls, none reported the success of blinding. Four RCTs used a placebo with ginseng flavor and the same appearance as the ginseng group.^[8,9,11,16]^ However, 4 RCTs did not note the flavor,^[10,13,14,17]^ and 2 RCTs did not report such details.^[12,15]^ Unblinding would lead to an overestimation of treatment effects, known as performance bias.

The therapeutic effects of ginseng may depend on the availability and amounts of various constituents in the preparation. The relevant details are sometimes missing in many publications. Daily dosage of included trials ranged from 200 to 3000 mg, but no studies have been done for the optimum dose for improving the menopausal symptom. When we analyzed the outcome direction and dosage, treatment time, dosage and time, and type of outcomes, there were no clear relationships between dose and treatment time for significant changes of various outcomes. The heterogeneities of trials prevent to show clear association. Various types of ginseng were tested compared with placebo with several different regimens. The difference in significance may come from the type of ginseng and treatment dosages. The dosage and frequency of ginseng used in the included trials maybe insufficient to generate a significant effect for biochemical variables. The dose-ranging studies comparing various and escalating amounts of ginseng to outcomes are needed to answer these questions.

Possible mechanisms of action of ginseng on menopausal women's health include hormonal effects similar to those of estrogen. Ginsenosides, which are thought to be the principle active components of red ginseng, have been shown to exert estrogen-like actions in several studies. The anti-angiogenic effects of ginsenoside Rb1 have been ascribed to its interaction with ERβ in vitro.^[19]^ Ginsenoside Rg3 increases nitric oxide (NO) production by increasing phosphorylation and expression of endothelial NO synthase mediated by ER-dependent PI3-kinase and adenosine monophosphate-activated protein kinase.^[20]^ Ginsenoside Re activates cardiac potassium channels through a nongenomic pathway through ERα.^[21,22]^ Ginsenoside Rh1 and Rb1 have been shown to have weak estrogenic activity in vivo.^[23,24]^ Ginseng can exert beneficial estrogenic properties either through directly binding to estrogen receptors such as genistein, daidzein, and resveratrol or indirectly activating ERs on the symptoms of menopause.^[25–27]^

Reports of AEs with ginseng are few, and these depend on the type of ginseng. Only 3 RCTs assessed AEs.^[8,10,11]^ One RCT reported no AEs from KRG.^[8]^ Vaginal bleeding was reported in 1 RCT.^[11]^ Another RCT showed that half of the participants (n = 181) reported AEs from ginseng without a significant difference in AEs between the 2 groups.^[10]^ Most of reported AEs are unlikely related with AEs of ginseng, for example, influenza or cold. Vaginal bleeding is probably related with ginseng because of anticoagulant effects of ginseng.^[28,29]^ There are no available studies with post-marketing surveillance of KRG or other types of ginseng. One review showed no evidence of AEs with normal doses of KRG or ginseng, but it also noted a lack of data on long-term use.

There are several important limitations in this study. We cannot be certain that our searches located all relevant RCTs, although strong efforts were made to retrieve all RCTs. Moreover, publication bias and poor reporting by clinical trials are major sources of bias. Several unpublished negative RCTs could distort the overall picture. Another possible bias is that 9 of the included trials were carried out in Korea,^[8,9,11–16]^ which is one of the regions that has been shown to produce largely positive results, and the generalization of these results to other countries might be limited. In addition to these limitations, the failure to follow the CONSORT guideline may lead to score a high ROB for a trial regardless of whether the trial was done well. It is notable that most studies were supported by a ginseng company, which may have introduced a degree of bias. Most of the industry-sponsored studies have been tended to report positive outcomes.^[30–32]^ Eight trials were funded by KRG (Korea Ginseng Corp),^[8,9,11,14–16]^ Bifido,^[12]^ or Pharmaton,^[10]^ and are opened to potential publication bias. Further limitations include the paucity and often suboptimal methodological quality of the primary data. Some of the RCTs included in the present review were not successful at minimizing bias. These issues limit the conclusiveness of this systematic review.

In conclusion, the existing trials showed positive effects of ginseng on sexual function, KRG for sexual arousal, and total hot flashes score in menopausal women. However, the included studies failed to show significant differences in hot flashes frequency, hormone levels, or endometrial thickness. The level of evidence is low owing to the small number of studies and the unclear ROB. Further rigorous RCTs are needed to overcome the many limitations of the current evidence.
